# Personal

**Published:** 1892-10

**Authors:** 


### Personal.

Dr. Rollin L. Banta, of Buffalo, has been appointed Professor of Materia Medica and Therapeutics in the Medical Department of Niagara University, vice Dr. A. E. Persons resigned. Dr. Banta's appointment is a gratification to his many friends, and will be advantageous to the Niagara School. We predict an interesting presentation of the subjects that Dr. Banta will teach.

